# Editorial: Recent Progresses of Non-coding RNAs in Biological and Medical Research

**DOI:** 10.3389/fgene.2020.00187

**Published:** 2020-02-28

**Authors:** Yun Zheng, Philipp Kapranov

**Affiliations:** ^1^Yunnan Key Lab of Primate Biomedicine Research, Institute of Primate Translational Medicine, Kunming University of Science and Technology, Kunming, China; ^2^Institute of Genomics, School of Biomedical Sciences, Huaqiao University, Xiamen, China

**Keywords:** non-coding RNA, long non-coding RNA (IncRNA), microRNA, biological research, medical research

## Introduction

Short (<200 nt) and long (>200 nt) non-coding (nc) RNAs account for majority of mammalian transcriptional output and encompass RNA species critical for various aspects of development and disease (Ambros, 2001; Kapranov et al., 2002, 2007; Bartel, 2004; Carninci et al., 2005). We have witnessed an ever-increasing pace of discovery of these transcripts in the last decade, in a large measure owing to the wide-spread application of high-throughput sequencing technologies for RNA analysis. These ncRNAs include, but not limited to, novel members of known classes such as miRNAs and siRNAs; new classes of small RNAs, for example, those associated with promoters and termini of genes; new classes of long non-coding (lnc) RNAs; plethora of antisense transcripts; circular RNAs derived from exons and introns; and many others (Laurent et al., 2015; Li et al., 2016; Kristensen et al., 2019; Zhang et al., 2019). Non-coding RNAs have been associated with almost every important biological process and human disease (Calin et al., 2004; Esteller, 2011; Wapinski and Chang, 2011; Mendell and Olson, 2012). However, our understanding of most of these transcripts is still at the initial stages.

Deeper insight into these enigmatic RNA species clearly requires efforts from both wet-lab and computational avenues of research (Zheng et al., 2017). Therefore, this Research Topic aimed to provide works from both directions to converge on generation of new insights into the functionalities of ncRNAs. Thirteen papers included in it serve as a collection of recent results and advances across multiple areas of ncRNA research field.

## Wet-lab Experimental Studies of ncRNAs

Lin et al. identified miR-30c secreted by bovine embryos as a potential biomarker for hampered preimplantation. Two miRNAs, i.e., miR-30c and miR-10b, were found at much higher levels in conditioned medium of slow cleaving embryos compared to intermediately cleaving ones (Lin et al.). One of them, miR-30c, directly repressed cyclin-dependent kinase 12 (CDK12) through a complementary site in the 3′ UTR (Lin et al.). Several DNA damage response (DDR) genes were significantly downregulated after introducing miR-30c or repressing CDK12, suggesting that miR-30c regulates embryo development through the DDR pathway (Lin et al.).

Mature hair follicles in mammals undergo periodic self-renewal processes called hair follicle cycles. Understanding the molecular regulatory mechanisms of the renewal cycle is important in medicine and developmental biology. Zhao et al. examined deregulated miRNAs, lncRNAs and circRNAs in the hair follicle cycle of Angora Rabbit (*Oryctolagus cuniculus*) and provides comprehensive repository of ncRNAs potentially relevant to this process.

Wang et al. profiled lncRNAs in the CD4^+^ T cells in the mouse model of acute asthma. They found 36 up- and 98 down-regulated lncRNAs in the disease compared with the control samples (Wang et al.). The potential functions of deregulated lncRNA were analyzed by performing miRNA binding analysis (Wang et al.).

It has been well-established that miRNAs work by guiding RNA-induced silencing complex (RISC) to their target RNA binding sites in cytoplasm (Bartel, 2004). However, a steady stream of evidence shows that some miRNAs localize and potentially function in nucleus (Place et al., 2008; Ritland Politz et al., 2009; Liu et al., 2018). Xun et al. proposed an efficient experimental method to find miRNA binding sequences in genomic DNA *in vivo*, thus potentially identifying miRNA binding sites in the regulatory regions of genes.

## Computational Studies of ncRNAs

Ou-Yang et al. proposed a novel method called two-side sparse self-representation (TSSR) for predicting lncRNA-disease associations. TSSR significantly outperformed other tested methods and identified some candidate lncRNA-disease associations (Ou-Yang et al.).

Zhang et al. proposed a method called CRlncRC2 for predicting associations between lncRNAs and cancers. More than four hundred cancer-related lncRNA candidates were identified, which were evaluated by examining the Lnc2Cancer database, reviewing literature, and performing statistical analysis of multiple relevant data sources containing information on mutations and differential gene expression in cancers (Zhang et al.). These results demonstrated that CRlncRC2 is an effective and accurate method for identification of cancer-related lncRNAs (Zhang et al.).

LncRNAs are assumed to realize their functions by interacting with other molecules, such as proteins, chromatin and other RNA species. Shen et al. proposed a new method for identifying lncRNA-protein interactions by employing Kernel Ridge Regression, based on Fast Kernel Learning (LPI-FKLKRR). LPI-FKLKRR demonstrated a superior performance compared with a series of other methods as judged by area under precision recall curve.

Huang et al. introduced a computational method to predict interactions between lncRNAs and miRNAs leveraging the information of expression profile data for these transcripts and the graph convolution technique. The proposed model is based on the assumption that the interaction between an lncRNA and a miRNA could be deciphered from their co-expression pattern. Compared with the conventional miRNA-target prediction algorithms based on sequence matching, their work presents a new approach to predict lncRNA:miRNA interactions.

Fukunaga et al. introduced a web server, called LncRRIsearch, for predicting lncRNA:lncRNA and lncRNA:mRNA interactions in human and mouse. The tissue-specific expression and cellular localization data of lncRNAs are integrated in this web server to explore tissue-specific or subcellular-localized lncRNA interactions (Fukunaga et al.).

## Reviews and Perspectives

Li and Liu summarizing recent evidences suggesting that coding and non-coding properties are inherent to both coding and non-coding transcripts. In other words, some lncRNAs and circRNAs could be used to produce short peptides, i.e., have coding capabilities. On the other hand, 3′ and 5′ UTRs of coding genes have non-coding functions such as recruiting RNA-binding proteins (Li and Liu).

Smith et al. reviewed the miRNAs and lncRNAs that play key roles in the initiation and progression of pediatric solid tumors. Pediatric tumors, due to lower mutation load compared with adult ones, are assumed to arise from mis-regulation of networks normally functioning during development at transcriptional level (Smith et al.). The authors summarized accumulating evidence of involvement of miRNAs and lncRNAs in the regulatory networks functioning during oncogenesis.

Watson et al. explored small RNAs in neurodegenerative diseases. This comprehensive review discusses roles of various small RNAs in multiple neurodegenerative diseases, including Alzheimer's, Parkinson's, multiple sclerosis, Amyotrophoic lateral sclerosis, and Huntington's disease.

Recent evidences show that ncRNAs, both miRNAs and lncRNAs, could serve as communication factors between cells (Bayraktar et al., 2017; Bär et al., 2019). Ramón y Cajal et al. proposed that the interactions between miRNAs and lncRNAs might contribute to the cell-type specific outcomes and to the determination of cell fate. In one model, miRNAs could be competitively sequestered by tissue-specific lncRNAs. In another context, miRNAs released to extracellular space as ligands could interact with lncRNAs in different organs as receptors to either sequester the miRNAs or induce degradation of the miRNAs or the lncRNAs.

## Summary

Non-coding RNAs have been associated with various biological processes and human diseases. These phenomena were further expanded and reviewed by several studies in this Research Topic. A number of wet lab and computational methods as well as database resources reported in the Topic should help to refine the connections between ncRNAs and diseases and identify the mechanisms of actions of the former, thus further contributing to the advancement of the ncRNA field.

## Author Contributions

YZ and PK conceived of the work and wrote the manuscript.

### Conflict of Interest

The authors declare that the research was conducted in the absence of any commercial or financial relationships that could be construed as a potential conflict of interest.
